# Long-range transport of radiocaesium derived from global fallout and the Fukushima accident in the Pacific Ocean since 1953 through 2017—Part I: Source term and surface transport

**DOI:** 10.1007/s10967-018-6244-z

**Published:** 2018-10-19

**Authors:** Michio Aoyama

**Affiliations:** Institute of Environmental Radioactivity, Fukushima University, Fukushima, 960-1296 Japan

**Keywords:** Radiocaesium, Source term, Fallout, Fukushima accident, Long range, Surface transport

## Abstract

Long range transport of radiocaesium derived from local fallout occurred early 1950s, global fallout which occurred mainly late 1950s and early 1960s and the Fukushima accident occurred in 2011 were investigated and presented for ocean surface in the Pacific Ocean. HAM database and its update were used in this study to present whole history of radioccaesium transport in surface layer in the interested region. Since both the main local/global fallout regions and injection of radiocaesium by Fukushima accident occurred in the western North Pacific and constrain of surface current systems which governed surface transport processes were subtropical gyre and subarctic gyre, radiocaesium transport in surface water in the mid latitude was characterized as rapid eastward transport along Kuroshio and Kuroshio extension. Behaviors were similar and repeated for local/global fallout and Fukushima derived radiocaesium. A part of radiocaesium transported/deposited/injected in the mid latitude subducted into ocean interior and the radiocaesium activity concentrations were kept higher rather than those in surface water.

**Electronic supplementary material:**

The online version of this article (10.1007/s10967-018-6244-z) contains supplementary material, which is available to authorized users.

## Introduction

The world’s oceans act as a sink for artificial radionuclides as well as for other anthropogenic pollutants released into the environment. Owing to physical and biogeochemical processes in the ocean, artificial radionuclides in the ocean are redistributed from their initial entry points which depend on the various sources. The extent of radioactive contamination in the ocean due to atmospheric weapons tests, releases from nuclear fuel reprocessing plants, and accidental releases from nuclear power plants (i.e., the Chernobyl accident in 1986 and the Fukushima accident in 2011) is of global concern [1–3].

Caesium-137 (^137^Cs) is one of the most abundant anthropogenic radionuclides in the marine environment with a half-life of 30.17 years. ^137^Cs has been released to the marine environment as a result of global and local fallout from atmospheric nuclear weapons tests [4], discharges from nuclear reprocessing plants, dumping of nuclear wastes into the global oceans, and fallout from the Chernobyl accident and the Fukushima Accident [5].

^137^Cs is one of the most useful tracers of water motion in the ocean, because its ocean input is well characterized; the major input of ^137^Cs in the ocean occurred as approximately a single injection, and the geographic distribution of this ^137^Cs injection has been re-constructed from fallout data and from ^137^Cs inventories in the soil and in water columns. The spatial and temporal changes of ^137^Cs in seawater reflect the flow of seawater such as through advection and diffusion, because most of the ^137^Cs exists in the dissolved form in seawater.

In terms of radioecology and environmental monitoring in marine environment, the main artificial contribution to the exposure of the world’s population has come from the atmospheric weapons tests and ^137^Cs and ^90^Sr are main contributors.

Therefore many researchers and governmental monitoring system focused their effort to ^137^Cs and ^90^Sr. As results, we have much number of observed ^137^Cs and ^90^Sr activity concentrations, temporal variation and horizontal and vertical distributions of ^137^Cs and ^90^Sr activities in marine environment as shown in the IAEA’s marine information system (MARiS) (https://maris.iaea.org/) and HAM database by Aoyama et al. [6] and its update. However, the sources of artificial and natural radionuclides found in the world’s oceans and systematic understanding of spatial and temporal change of ^137^Cs and ^90^Sr activities have not been comprehensively studied, despite the importance of such knowledge. Among the major long-lived artificial and natural radionuclides in the marine environment, ^3^H, ^14^C, ^90^Sr, ^134^Cs, ^137^Cs, and plutonium isotopes were released by atmospheric nuclear weapons tests and ^134^Cs and ^137^Cs were released by the two major nuclear power plant accidents at Chernobyl and Fukushima especially from Fukushima accident in the Pacific Ocean. In addition, ^3^H and ^14^C are produced naturally in the stratosphere by cosmic rays [2].

In this article, the author show source term of radiocaesium in the Pacific Ocean and the behavior of long range transport of radiocaesium, mainly for ^137^Cs, in surface water since 1954 until 2016 to understand a complete history of radiocaesium contamination in the Pacific Ocean. The author summarized a feature of global fallout, local fallout at Pacific Providing Ground, fallout/injection from the Chernobyl accident and the Fukushima accident. Then full deposition history in Japan since 1945 until 2016 to show temporal change of source term of ^137^Cs to the Pacific Ocean mainly by global fallout, the Chernobyl accident and the Fukushima accident. Temporal changes of horizontal distribution of ^137^Cs activity concentration in surface water in the Pacific Ocean are presented since 1954 until 2016. In the last section of the results and discussion, processes of surface to ocean interior transport of radiocaesium are shown and discussed both in case of Fukushima accident and global fallout to show importance of subduction which affected much on the behaviors of radiocaesium in the Pacific Ocean.

### Key feature of global fallout

The atmospheric testing of nuclear weapons, which was carried out from 1945 to 1980 (Table 1), is the largest source of the artificial radioactivity presently in the marine environment. The United States, for example, conducted an extensive campaign, grouped into roughly 20 test series, of atmospheric nuclear tests from 1945 to 1963. In 1963, the Limited Test Ban Treaty was signed, and testing by the United States, the Soviet Union, and Great Britain moved underground, but France continued atmospheric testing until 1974 and China continued until October 1980, respectively. Numbers of atmospheric weapons tests in each year and annual total yield of fission and fusion yields from atmospheric testing by all countries are summarized in Table 1. The majority of tests were conducted during 1951–1958 and 1961–1962. A moratorium was observed in 1959, and largely observed in 1963. The most active years of testing from the standpoint of the total explosive yields were 1962, 1961, 1958, and 1954 in descending order. The total number of atmospheric tests conducted by all countries was 543, and the total yield was 440 Mt. The fission yield of all atmospheric tests is estimated to be 189 Mt [2].

**Table 1:** Number of tests of each year and annual total yield of fission and fusion yields of nuclear tests, all countries. Reproduced and summarized from Table 4 in United Nations, *“ANNEX C”,* in *Sources and Effects of Ionizing Radiation. United Nations Scientific Committee on the Effects of Atomic Radiation, 2000 Report to the General Assembly, with scientific Annexes*, **I**, United Nations publication, Austria, 2000, 158–291 (2000)]

Year	Number of tests	Total yield (Mt)
1945	3^a^	0.057
1946	2	0.042
1947		
1948	3	0.1
1949	1	0.022
1950		
1951	18	0.59
1952	11	11
1953	18	0.71
1954	16	48.3
1955	20	2.06
1956	32	22.9
1957	46	9.64
1958	91	56.8
1959		
1960	3	0.072
1961	59	86.5
1962	118	170.4
1963		
1964	1	0.02
1965	1	0.04
1966	8	1.14
1967	5	3.18
1968	6	7.6
1969	1	3
1970	9	5.78
1971	6	1.46
1972	5	0.13
1973	6	2.52
1974	8	1.21
1975		
1976	3	4.12
1977	1	0.02
1978	2	0.04
1979		
1980	1	0.6
Total	543^b^	440

^a^Includes two cases of military combat use in Japan

^b^Total includes additional 39 safety tests: 22 by the United States, 12 by the United Kingdom, and 5 by France

**Fig. 1:**
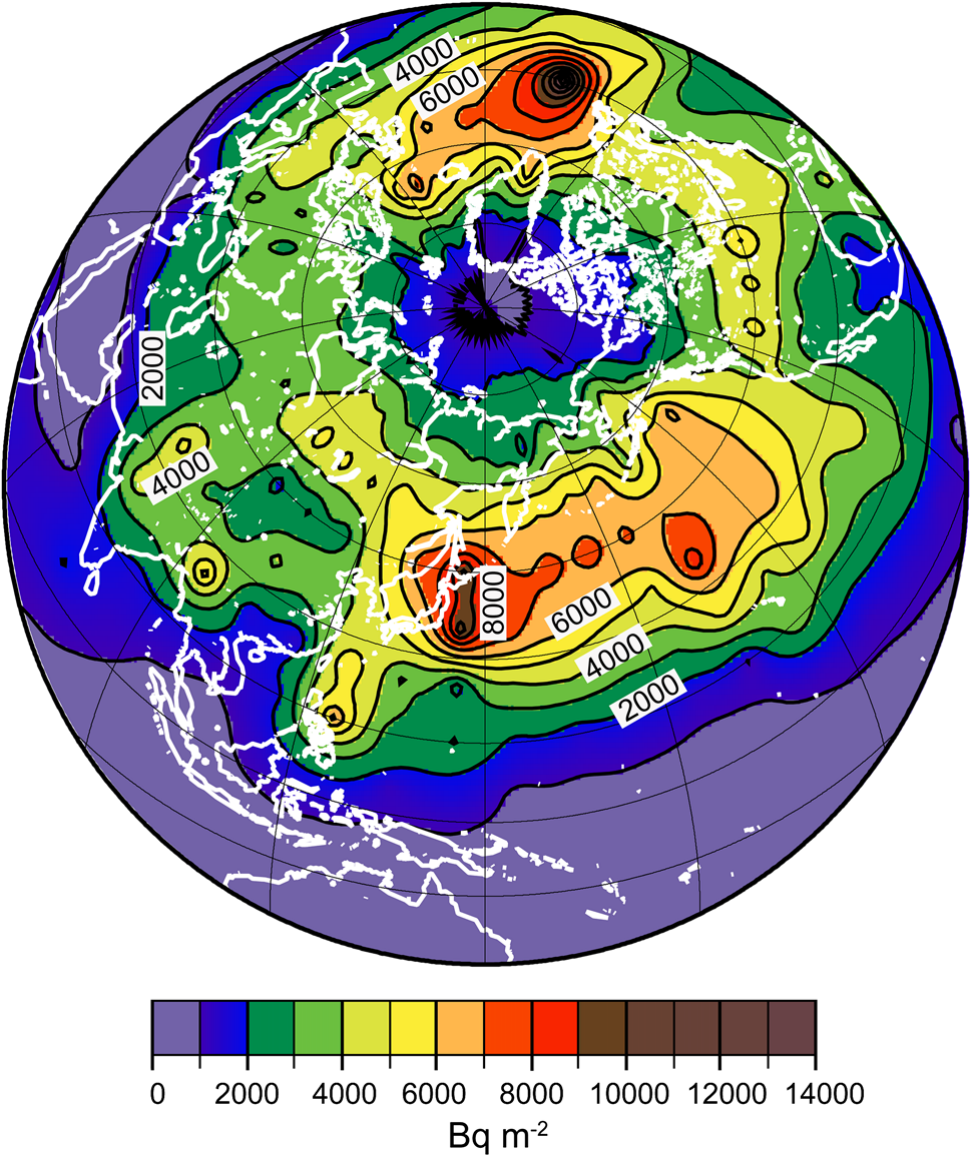
Global distribution of ^137^Cs fallout as of 1970 (reproduced from Fig. 1 in [4])

The global spatial distribution of ^137^Cs fallout, based on measurements of ^137^Cs in rainwater, seawater, and soil, have been precisely reported as 10° × 10° gridded data [4]. The geographical distribution of global fallout in the Northern Hemisphere is characterized by two regions of high ^137^Cs fallout (Fig. 1), one in the Kuroshio Current and Kuroshio extension areas (latitude 20–40°N) of the Pacific Ocean, and the other corresponding to the area affected by the Gulf Stream (latitude 30–50°N) in the Atlantic Ocean. These regions are characterized by both high precipitation amounts and higher stratosphere–troposphere exchange rates. ^137^Cs was injected into the stratosphere by nuclear weapons tests, then stratosphere–troposphere exchange is the main process of ^137^Cs which contribute transport process of ^137^Cs to the troposphere, and precipitation scavenging is the main process by which ^137^Cs in the troposphere is deposited on the Earth’s surface. In the Northern Hemisphere, stratosphere–troposphere exchange occurs mainly in the latitude band from 40° to 70°N, and regional maxima are observed east of the North American and Asian continents and in Europe. The Northern Hemisphere precipitation pattern reflects heat and freshwater fluxes from the equator to the Arctic region along the Kuroshio Current and the Gulf Stream. As a result, the twin high global ^137^Cs fallout regions are located where these two patterns intersect [4].

**Fig. 2:**
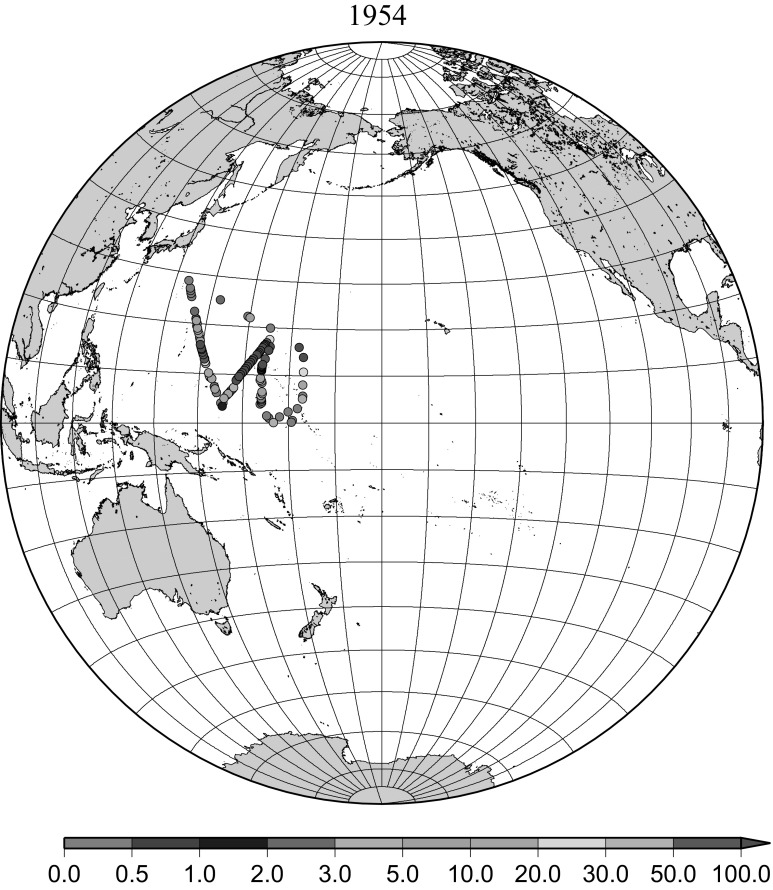
Horzontal distribution of ^137^Cs activity concentration in the North Pacific in 1954. Unit: Bq m^−3^

### Pacific providing ground (PVG) test including castle tests at Bikini atoll in early 1950s

**Fig. 3:**
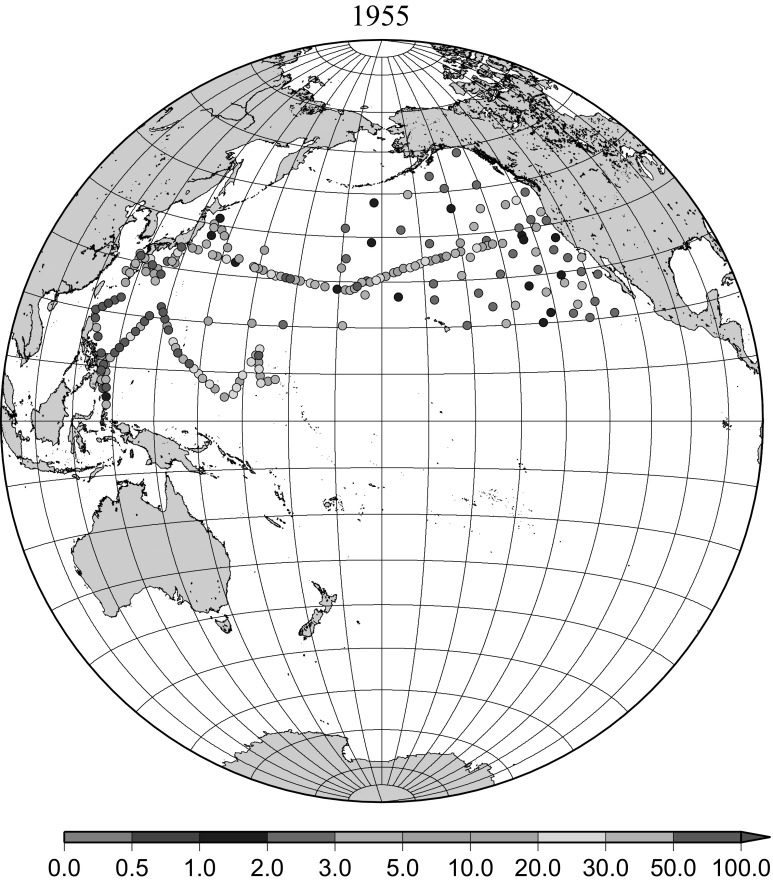
Horzontal distribution of ^137^Cs activity concentration in the North Pacific in 1955. Unit: Bq m^−3^

A large scale contamination of the ocean surface in the western North Pacific Ocean originated from ground tests of nuclear devices at Bikini atoll called “Castle Bravo” and from a longer series of tests of various devices in the same region in May and June 1954. The first Shunkotsu-maru expedition was undertaken about 1 month after the Castle test [7, 8]. In the surface seawater, maximum activity of more than 1000 dpm L^−1^ was distributed up to 2000 km WNW of the test site along the North Equatorial Current. From February to May 1955, the highest activity was located off the coast of Luzon Island in the Philippines where the activity was 570 dpm L^−1^ and some activity still remained in the water along the North Equatorial Current [9]. In summer 1955, a joint expedition by Canada, Japan and United States found a slight radioactivity concentration, 0–30 dpm L^−1^, but widespread radioactivity in the large part of the western North Pacific [10]. The maximum activity was observed along the Kuroshio Current south of Japan in summer 1955. In this study the radioactivity concentration reported in terms of total decay count per minute as dpm L^−1^ [7–11] were converted to ^90^Sr activity concentration, then converted ^137^Cs activity concentration were used in the Figs. 2 and 3 and a part of data in Fig. 5. Figure 2 shows the radioactive contamination within 2 months of the test of the nuclear devices at Bikini atoll in May 1954. This clearly shows the pathway of the contaminated areas along the North Equatorial Current in the subtropical gyre in the North Pacific Ocean. Figure 3 shows areas of radioactive contamination in 1955 and the pathway of the contaminated areas along the North Equatorial Current and Kuroshio Current in the subtropical gyre in the North Pacific Ocean, but not relatively high radioactivity concentration in the eastern North Pacific Ocean.

**Fig. 4:**
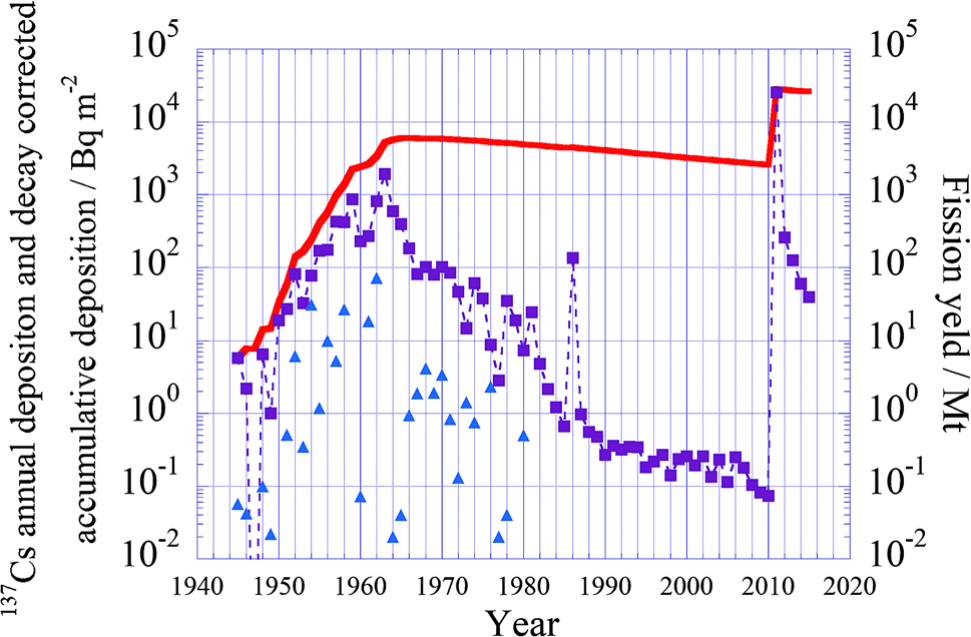
Deposition history at Tokyo/Tsukuba since 1945 until 2016

**Fig. 5:**
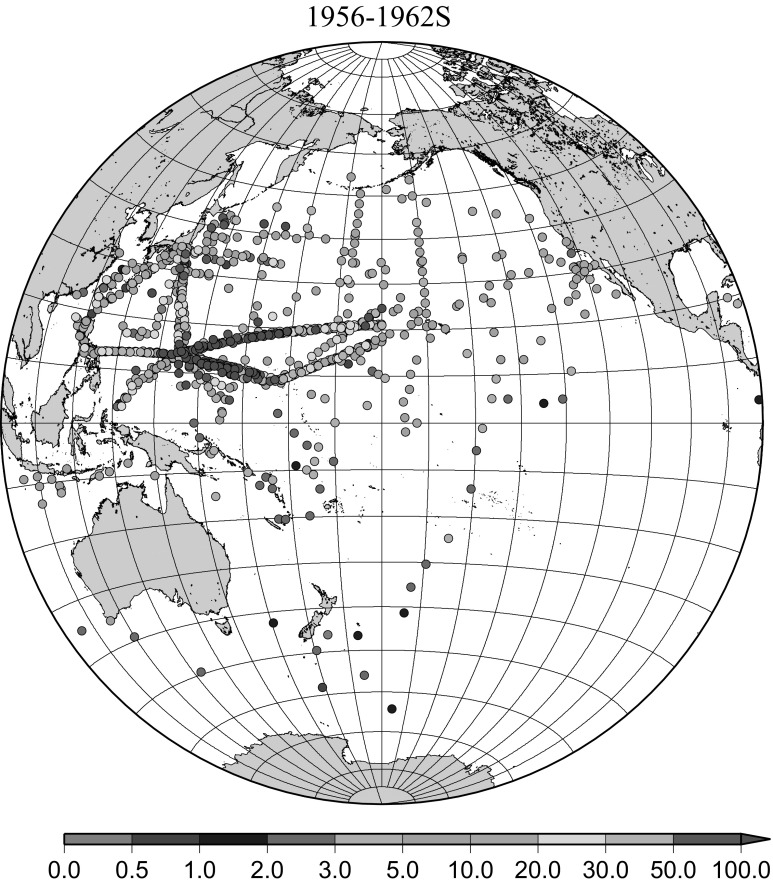
Horzontal distribution of ^137^Cs activity concentration in the North Pacific in 1956–1962. Unit: Bq m^−3^

### Chernobyl accident in 1986

On 26 April 1986, an explosion and fire at the Chernobyl Nuclear Power Plant (ChNPP) in Ukraine caused the largest uncontrolled radioactive release in the history of the civil nuclear industry, and large quantities of radioactive iodine and cesium were released into the air due to the explosion and fire at the accident site. Most of this radioactive material was deposited near the installation, but a substantial amount of these radionuclides was quickly carried by wind over Ukraine, Belarus, the territory of the present-day Russian Federation, and, to some extent, parts of Europe within a week. Thereafter, Chernobyl radionuclides spread globally, and they reached East Asia by around 3 May 1986, just 1 week after the accident [12].

Because most of the radioactivity released by the ChNPP accident was deposited on land, little attention was initially paid to the impact of the released radioactivity on the marine environment, even though the total release of radionuclides exceeded the total release by the Fukushima accident by an order of magnitude. This lack of attention can be explained by the fact that most of the radioactivity released by the ChNPP accident was deposited on land. The maximum radionuclide activity concentrations in marine environments at the Baltic and Black seas following the ChNPP accident were smaller by several orders of magnitude than the maximum activity concentration in the coastal area around Fukushima following the accident there. However, because the areas of the Baltic and Black seas are small compared with the area of the North Pacific Ocean, radiocaesium concentrations in these two seas were higher during the initial post-accident period than concentrations in most of the North Pacific, excepting the coastal waters off Fukushima. In addition, radionuclide transfer processes in the marine environment differed between the ChNPP and Fukushima events because of differences in (1) source intensity, (2) marine basin geometry, (3) ocean circulation, (4) bottom sediment composition, and (5) food chains of marine organisms between the Baltic and Black seas and the North Pacific [13].

### Fukushima accident in March 2011, Japan

The Fukushima Daiichi Nuclear Power Station (FNPPl; 37.42°N, 141.87°E) is in Futaba, Fukushima Prefecture, Pacific coast of Japan. On 11 March 2011, an extraordinary earthquake of magnitude 9.0 centered at 38.3°N, 142.4°E, about 130 km east off the Pacific coast of Japan’s main island of Honshu, was followed by a huge Tsunami with waves that reached heights of up to 40 m in Iwate Prefecture and up to about 10 m in Fukushima Prefecture [14]. One consequence of the Tsunami was a station blackout (total loss of AC electric power) at FNPP1, which led to a meltdown of three reactors [5] and the discharge of substantial amount of radionuclides into both the air and ocean [15, 16]. The largest and earliest source of artificial radionuclides to the environment from the FNPP1 accident was the atmospheric release, which peaked in mid-March 2011. The total amount of atmospheric release of ^137^Cs was estimated to be 15.2–20.4 PBq, and the same amount of ^134^Cs was released because the ^134^Cs/^137^Cs activity ratio was close to 1. About 20% of the released radiocaesium was deposited on land, and the other 80% was deposited onto the sea surface. As a result, the North Pacific Ocean received 11.7–14.8 PBq of ^137^Cs as atmospheric deposition. The direct discharge of contaminated waters to the ocean started on 26 March and peaked on 6 April 2011, as inferred by an analysis of ^131^I/^137^Cs activity ratios [17]. The total amount of ^137^Cs directly released to the ocean is estimated to be 3.5 ± 0.7 PBq [16, 17]. Therefore, the combined input of ^137^Cs to the North Pacific Ocean by both atmospheric deposition and direct discharge is estimated to be 15.2–18.3 PBq [16, 18–20] (Table 2).

**Table 2:** Data sources of Figs. 2, 3, 5, 6, 7, 8, 9, 10, 11, 12, 13, 14, 15, 16, 17, 18, 19, 20, 21 and 22

MRIREFNO	Begin	End	1954	1955	1956–1962	1963–1969	1970s	1980s	1986	1990s	2000s	2011	2012	2013–2014	2015–2016
Shunnkotu_1stCruise	1954	1954	x*												
NORPAC&Operation troll	1955	1955		x*											
Pacific Sea Water samples and warship Walton	1956	1958			x*										
Miyake1960	1956	1959			x										
Miyake1961	1957	1959			x										
Saruhashi1975	1957	1973			x	x	x								
Miyake1962	1958	1960			x										
Miyake1963	1958	1960			x										
Folsom1960a	1959	1960			x										
Folsom1960b	1960	1960			x										
Folsom1968	1960	1967			x	x									
Broecker1966	1963	1963				x									
Nagaya1965	1963	1964				x									
Broecker1968	1963	1966				x									
Nagaya1970	1963	1968				x									
Pillay1964	1964	1964				x									
Wong1971	1964	1964				x									
Shirasawa1968	1964	1967				x									
MSA1965-2010	1965	2010				x	x	x	x	x	x				
Folsom1970	1965	1968				x									
Folsom1979	1967	1972				x	x								
Nagaya1976	1969	1973				x	x								
Katsuragi_unpublish	1971	1972					x								
Noshkin1974a	1972	1972					x								
Noshkin1974b	1972	1972					x								
Noshkin1999	1972	1983					x								
Folsom1975	1973	1973					x								
Livingston1985	1973	1974					x								
Noshkin1976	1975	1975					x								
Miyake1988	1975	1978					x								
Nagaya1981	1976	1979					x								
Noshkin2000	1976	1983					x	x							
Noshkin1978	1977	1977					x								
Domanov1984	1978	1978					x								
Livingston2000	1978	1978					x								
Noshkin1987	1978	1978					x								
Livingston1984	1979	1979					x								
Aoyama1995	1979	1987					x	x							
Bowen1982	1980	1980						x							
Nagaya1984	1980	1982						x							
Hirose1992	1981	1981						x							
Hirose2000c	1981	1987						x	x						
MERI1985-2016	1984	1984						x	x	x	x	x	x	x	x
Nagaya1987	1984	1985						x							
Hirose2002	1986	1987						x	x						
Wong1992	1986	1987						x	x						
Hirose2001	1987	1997						x		x					
Nagaya1993	1988	1988						x							
Yamada2007	1992	1992								x					
Bourlat1996	1993	1993								x					
Hong1999	1993	1993								x					
Kang1997	1993	1993								x					
Miyao1998	1993	1993								x					
Yamada1996	1993	1993								x					
JKRJE1995	1994	1994								x					
Hirose1999	1995	1995								x					
Ikeuchi1999	1995	1995								x					
Yamada2006	1996	1997								x					
BSH1997	1997	1997								x					
Yamada1997	1997	1997								x					
Aoyama2001	1997	1998								x					
Ito2003	1997	2000								x	x				
Delfanti2000	1998	1998								x					
Povinec2003	1998	1998								x					
Ito2005	2001	2002									x				
Aoyama2008	2002	2002									x				
Domanov2004	2002	2002									x				
Aoyama2011	2003	2003									x				
Povinec2011	2004	2004									x				
Inoue2011	2009	2011									x	x			
Busseler2012	2011	2011										x			
CKKim2012	2011	2011										x			
JAMSTEC2011_F	2011	2011										x			
Aoyama2013	2011	2012										x			
Kaeriyama2013	2011	2012										x	x		
Kamenik2013	2011	2012										x			
KINS2012-2016	2011	2012										x	x	x	x
Kumamoto2015	2011	2012										x			
Suseno2015	2011	2012										x	x		
Kaeriyama2013b	2011	2013										x	x		
Suseno2014	2011	2013										x	x	x	
Aoyama2016	2011	2014										x	x	x	
Zhou2018	2011	2014										x	x	x	
Kumamoto2017	2012	2012											x		
Aoyama2018	2015	2017													x
Aoyama_this study	1986	2007						x	x	x	x				

X*: activity is converted from total beat count

X: activity is observed and reported activity at the time of sampling

### Full deposition history since 1945 at Tokyo/Tsukuba, Japan

Annual deposition of ^137^Cs and decay corrected accumulative deposition since 1945 until 2016 are shown in Fig. 4. Data sources of annual deposition are Aoyama, 1999 [21], http://www.mri-jma.go.jp/Dep/ap/ap4lab/recent/ge_report/2015Artifi_Radio_report/index.html as of 24 August 2018 and http://search.kankyo-hoshano.go.jp/servlet/search.top?pageSID=93755933, as of 27 August 2018. The total fission yield of atmospheric weapons test during the period from 1945 to 1980 in each year are also shown in Fig. 4. In terms of decay corrected accumulative deposition of ^137^Cs which corresponds total inventory of ^137^Cs in soil and water column in the ocean if we assume ^137^Cs does not move from deposited place. In fact it can be close to reality on land, but in the ocean ^137^Cs easily moved due to ocean current and subduction/obduction.

**Table 3:** Decay corrected cumulative deposition and annual deposition in Tokyo until 1980 and Tsukuba since 1980–2016. Unit: Bq m^−2^

Year	Decay corrected cumulative deposition	Annual deposition	Source of deposition data
1945	5.78E+00	5.78E+00	Aoyama 1999
1946	7.85E+00	2.20E+00	Aoyama 1999
1947	7.67E+00	0.00E+00	Aoyama 1999
1948	1.40E+01	6.53E+00	Aoyama 1999
1949	1.47E+01	1.01E+00	Aoyama 1999
1950	3.30E+01	1.86E+01	Aoyama 1999
1951	5.92E+01	2.70E+01	Aoyama 1999
1952	1.38E+02	8.06E+01	Aoyama 1999
1953	1.68E+02	3.24E+01	Aoyama 1999
1954	2.41E+02	7.73E+01	Aoyama 1999
1955	4.06E+02	1.70E+02	Aoyama 1999
1956	5.71E+02	1.74E+02	Aoyama 1999
1957	9.85E+02	4.27E+02	Aoyama 1999
1958	1.38E+03	4.22E+02	Aoyama 1999
1959	2.22E+03	8.72E+02	Aoyama 1999
1960	2.40E+03	2.29E+02	Aoyama 1999
1961	2.62E+03	2.72E+02	Aoyama 1999
1962	3.37E+03	8.11E+02	Aoyama 1999
1963	5.23E+03	1.93E+03	Aoyama 1999
1964	5.70E+03	5.97E+02	Aoyama 1999
1965	5.97E+03	3.92E+02	Aoyama 1999
1966	6.01E+03	1.83E+02	Aoyama 1999
1967	5.96E+03	8.07E+01	Aoyama 1999
1968	5.92E+03	1.02E+02	Aoyama 1999
1969	5.87E+03	7.95E+01	Aoyama 1999
1970	5.84E+03	1.02E+02	Aoyama 1999
1971	5.79E+03	8.41E+01	Aoyama 1999
1972	5.70E+03	4.60E+01	Aoyama 1999
1973	5.59E+03	1.46E+01	Aoyama 1999
1974	5.52E+03	6.11E+01	Aoyama 1999
1975	5.43E+03	3.73E+01	Aoyama 1999
1976	5.32E+03	8.84E+00	Aoyama 1999
1977	5.22E+03	2.82E+00	Aoyama 1999
1978	5.14E+03	3.49E+01	Aoyama 1999
1979	5.04E+03	1.87E+01	Aoyama 1999
1980	4.93E+03	7.36E+00	Aoyama 1999
1981	4.84E+03	2.41E+01	Aoyama 1999
1982	4.74E+03	4.81E+00	Aoyama 1999
1983	4.63E+03	2.15E+00	Aoyama 1999
1984	4.53E+03	1.22E+00	Aoyama 1999
1985	4.43E+03	6.66E−01	Aoyama 1999
1986	4.46E+03	1.35E+02	Aoyama 1999
1987	4.36E+03	9.62E−01	Aoyama 1999
1988	4.26E+03	5.55E−01	Aoyama 1999
1989	4.16E+03	4.74E−01	Aoyama 1999
1990	4.07E+03	2.70E−01	Aoyama 1999
1991	3.98E+03	3.57E−01	Aoyama 1999
1992	3.89E+03	3.20E−01	Aoyama 1999
1993	3.80E+03	3.46E−01	Aoyama 1999
1994	3.71E+03	3.43E−01	Aoyama 1999
1995	3.63E+03	1.81E−01	Aoyama 1999
1996	3.55E+03	2.18E−01	Aoyama 1999
1997	3.47E+03	2.69E−01	Aoyama 1999
1998	3.39E+03	1.41E−01	Government monitoring database
1999	3.31E+03	2.34E−01	Government monitoring database
2000	3.24E+03	2.55E−01	Government monitoring database
2001	3.16E+03	1.92E−01	Government monitoring database
2002	3.09E+03	2.58E−01	Government monitoring database
2003	3.02E+03	1.35E−01	Government monitoring database
2004	2.95E+03	2.33E−01	Government monitoring database
2005	2.89E+03	1.14E−01	Government monitoring database
2006	2.82E+03	2.50E−01	Government monitoring database
2007	2.76E+03	1.79E−01	Government monitoring database
2008	2.69E+03	1.04E−01	Government monitoring database
2009	2.63E+03	8.17E−02	Government monitoring database
2010	2.57E+03	7.34E−02	Artificial radioactivity in the environment 2015
2011	2.80E+04	2.55E+04	Artificial radioactivity in the environment 2015
2012	2.77E+04	2.60E+02	Government monitoring database
2013	2.72E+04	1.26E+02	Government monitoring database
2014	2.66E+04	5.99E+01	Government monitoring database
2015	2.60E+04	3.93E+01	Government monitoring database
2016	2.55E+04	3.71E+01	Government monitoring database

Unit Bq m^−2^

Aoyama 1999: Aoyama, M., Geochemical studies on behaviour of anthropogenic radionuclides in the atmosphere, Ph. D. thesis, Kanazawa Univ., Kanazawa, Japan

Artificial radioactivity in the environment 2015: http://www.mri-jma.go.jp/Dep/ap/ap4lab/recent/ge_report/2015Artifi_Radio_report/index.html as of 24 August 2018 government monitoring database and http://search.kankyo-hoshano.go.jp/servlet/search.top?pageSID=93755933, as of 27 August 2018

Main contributor to current existing ^137^Cs in our environment was large atmospheric weapons test late 1950s and early 1960s of which fission yields was ranged from 10 to 100 Mt as shown in Table 1 and Fig. 4. More than 90% of current existing ^137^Cs in our environment was produced during that period. At Tsukuba, due to long distance from Chernobyl accident site, annual deposition in 1986 was only 135 Bq m^−2^ which corresponded to ca. 3% of decay corrected accumulative deposition in 1985 [22, 23]. In contrast of Chernobyl accident, due to short distance between Tsukuba and Fukushima, annual deposition at Tsukuba was 25.5 kBq m^−2^ in 2011 which corresponded to about one order of magnitude larger than decay corrected accumulative deposition of 3 kBq m^−2^ in 2010. Annual deposition at Tsukuba after 2011 decreased [24].

### HAM database and its update used in this study

To establish database for artificial radionuclides in the marine environment is important to understand impact of artificial radionuclides to human. In 2004, the database “Historical Artificial Radionuclides in the Pacific Ocean and its Marginal Seas”, or HAM database, has been created and published [6]. The database includes ^90^Sr, ^137^Cs, and ^239,240^Pu activity concentration data from the seawater of the Pacific Ocean and its marginal seas with some measurements from the sea surface to the bottom. The data in the HAM database were collected from about 90 literature citations, which include published papers; annual reports by the Hydrographic Department, Maritime Safety Agency, Japan; and unpublished data provided by individuals. The data of concentrations of ^90^Sr, ^137^Cs, and ^239,240^Pu have been accumulating since 1957–1998. The HAM database included 7737 records for ^137^Cs concentration data, 3972 records for ^90^Sr concentration data, and 2666 records for ^239,240^Pu concentration data. The spatial variation of sampling stations in the HAM database is heterogeneous, namely, more than 80% of the data for each radionuclide is from the Pacific Ocean and the Sea of Japan, while a relatively small portion of data is from the South Pacific [6]. The data in this HAM database is already included in the IAEA’s Marine Information System (MARiS) (https://maris.iaea.org/). In this study the data in the HAM database was used and recently updated data for new HAM database for global version (Aoyama in preparation) was also used to draw figures in this article. The number of the literatures was increased from 90 to 157 and the data of activity concentrations of ^90^Sr, ^137^Cs, and ^239,240^Pu have been accumulating since 1953–2016. All of the 157 literatures are listed in Table S1. The new HAM database included 55,811 records for ^137^Cs concentration data, 19,076 records for ^90^Sr concentration data, and 7742 records for ^239,240^Pu concentration data. A part of new data is come from coastal region monitoring very close to Fukushima accident site.

## Results and discussion

### Results on horizontal distribution in surface water since 1954 until 2016

**Fig. 6:**
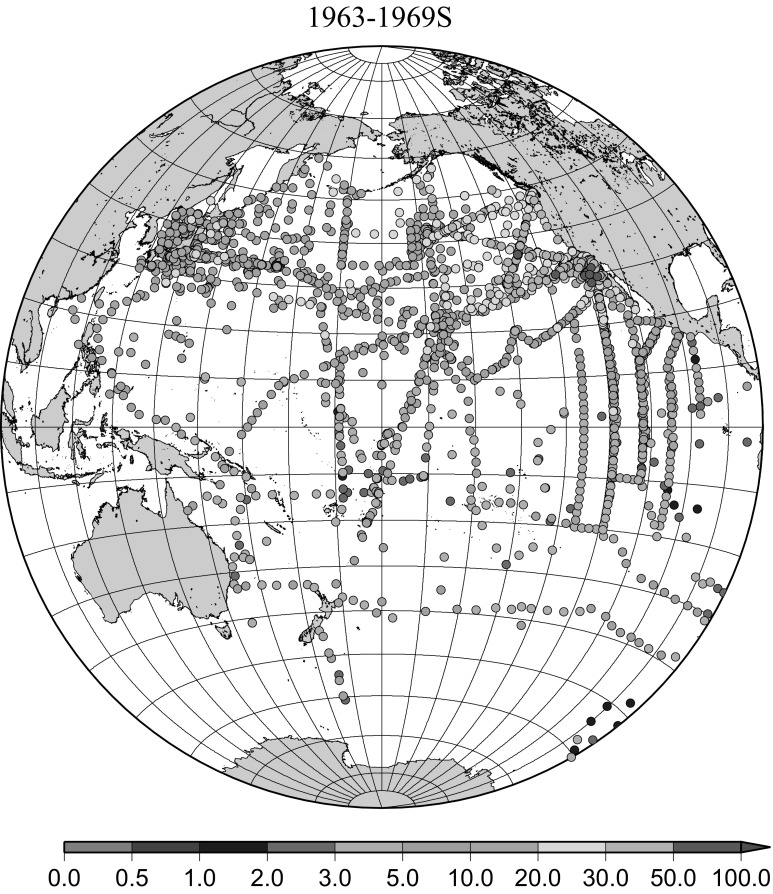
Horzontal distribution of ^137^Cs activity concentration in the North Pacific in 1963–1969. Unit: Bq m^−3^

**Fig. 7:**
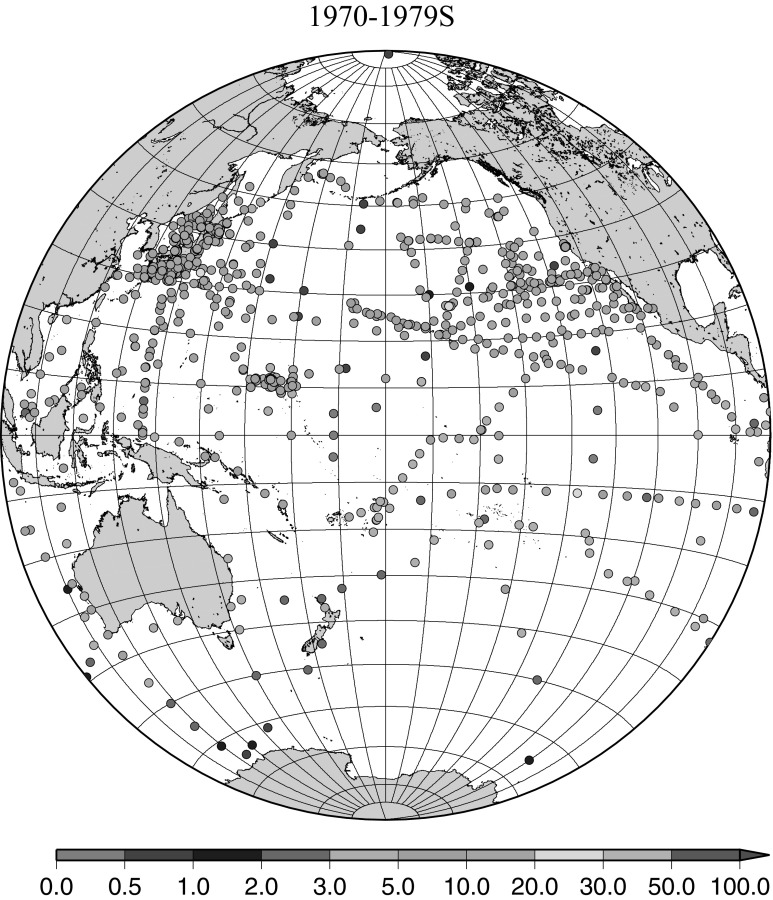
Horzontal distribution of ^137^Cs activity concentration in the North Pacific in 1970–1979. Unit: Bq m^−3^

**Fig. 8:**
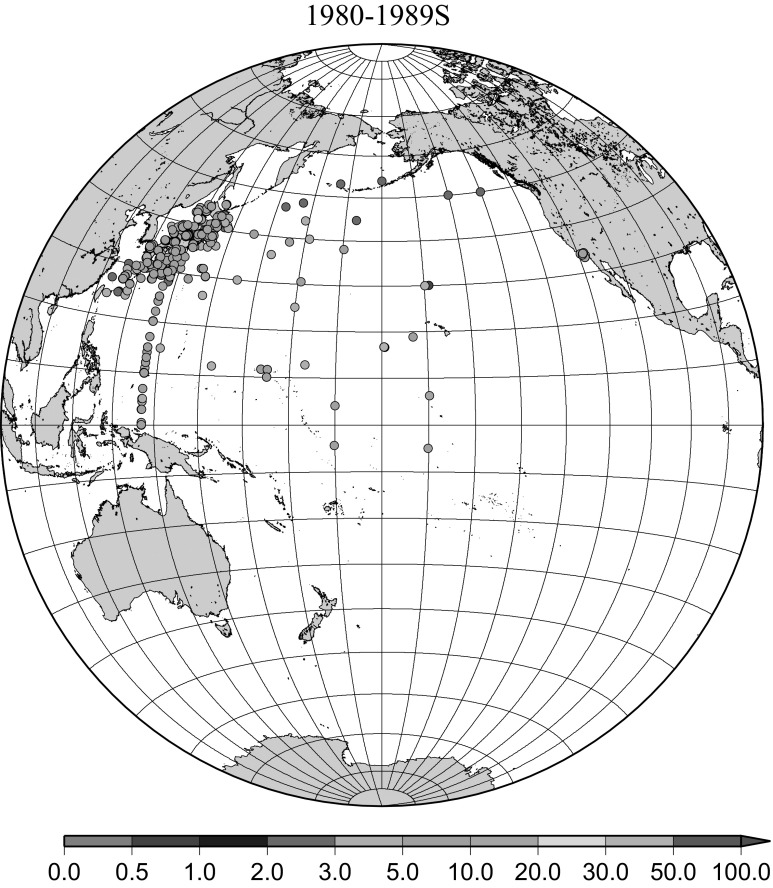
Horzontal distribution of ^137^Cs activity concentration in the North Pacific in 1980–1989. Unit: Bq m^−3^

**Fig. 9:**
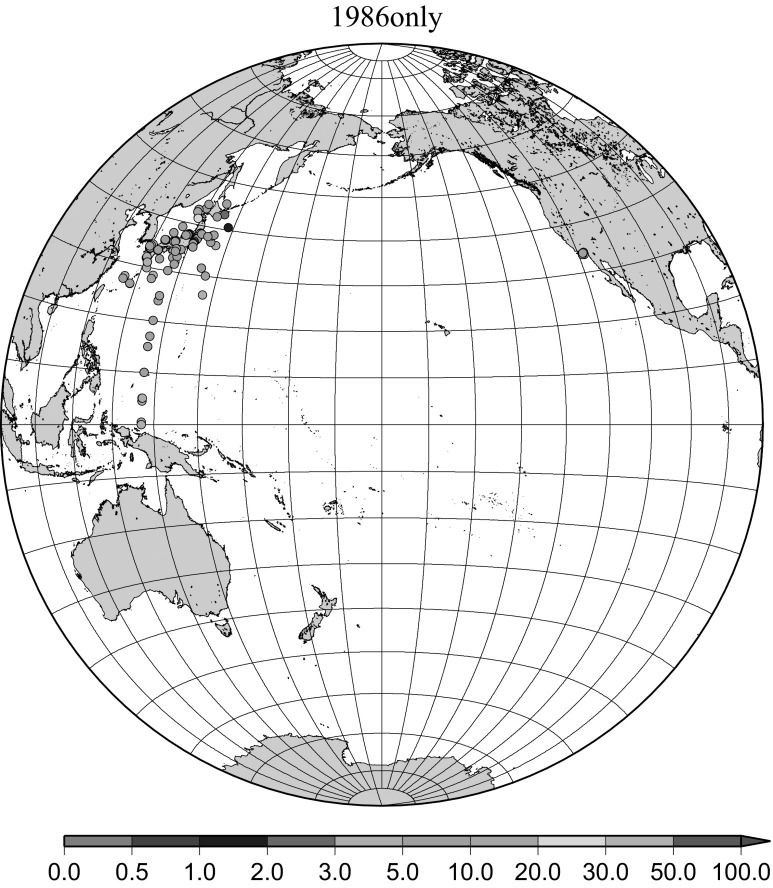
Horzontal distribution of ^137^Cs activity concentration in the North Pacific in 1986 only. Unit Bq m^−3^

**Fig. 10:**
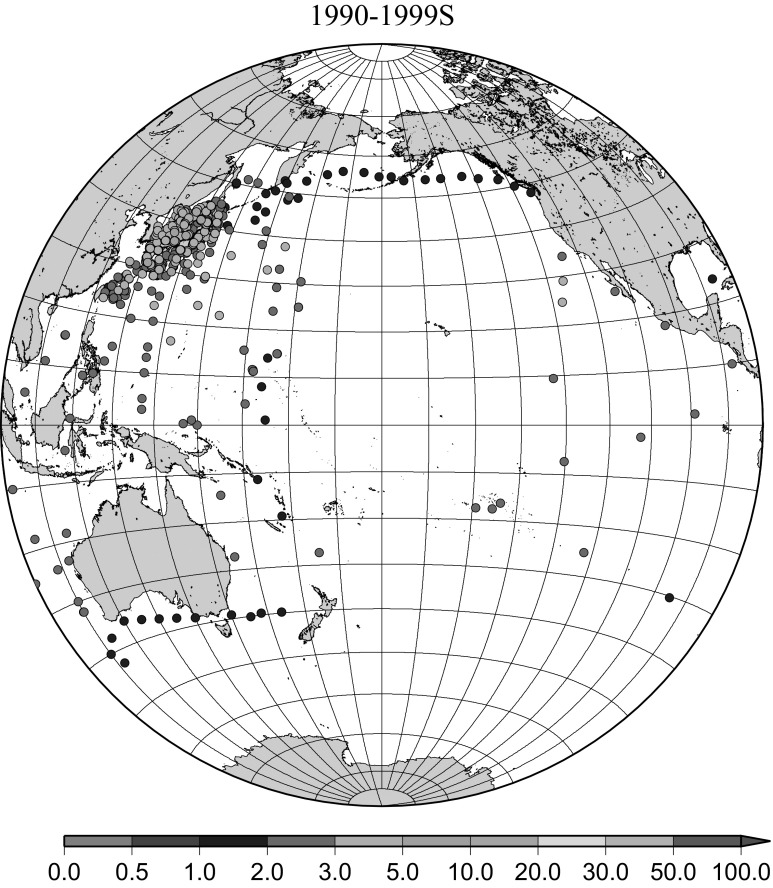
Horzontal distribution of ^137^Cs activity concentration in the North Pacific in 1990–1999. Unit: Bq m^−3^

**Fig. 11:**
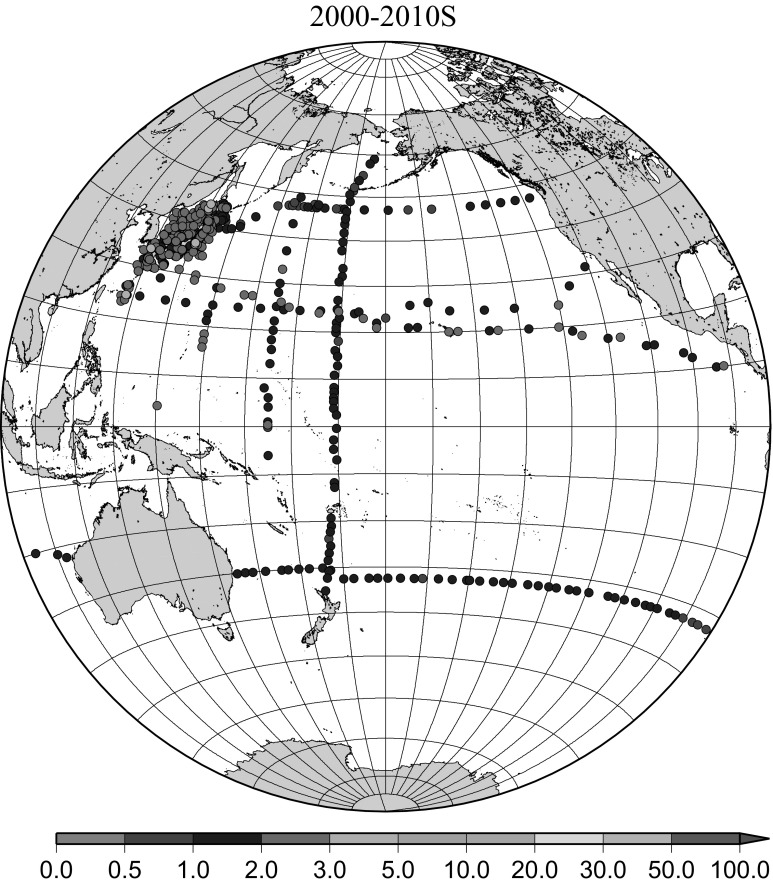
Horzontal distribution of ^137^Cs activity concentration in the North Pacific in 2000–2010. Unit: Bq m^−3^

**Fig. 12:**
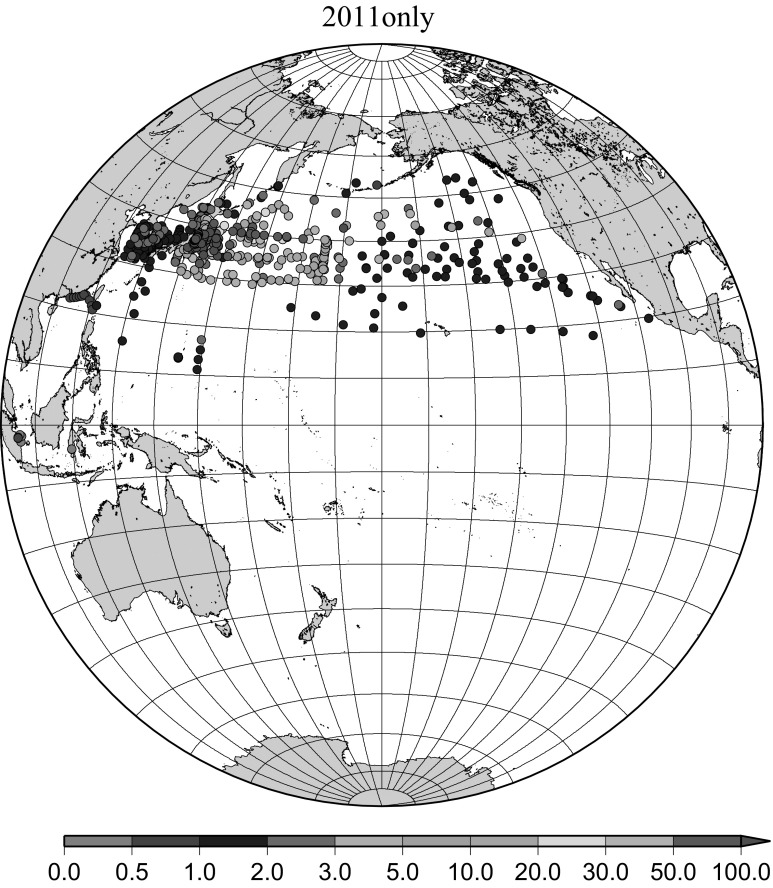
Horzontal distribution of ^137^Cs activity concentration in the North Pacific in 2011 only. Unit: Bq m^−3^

**Fig. 13:**
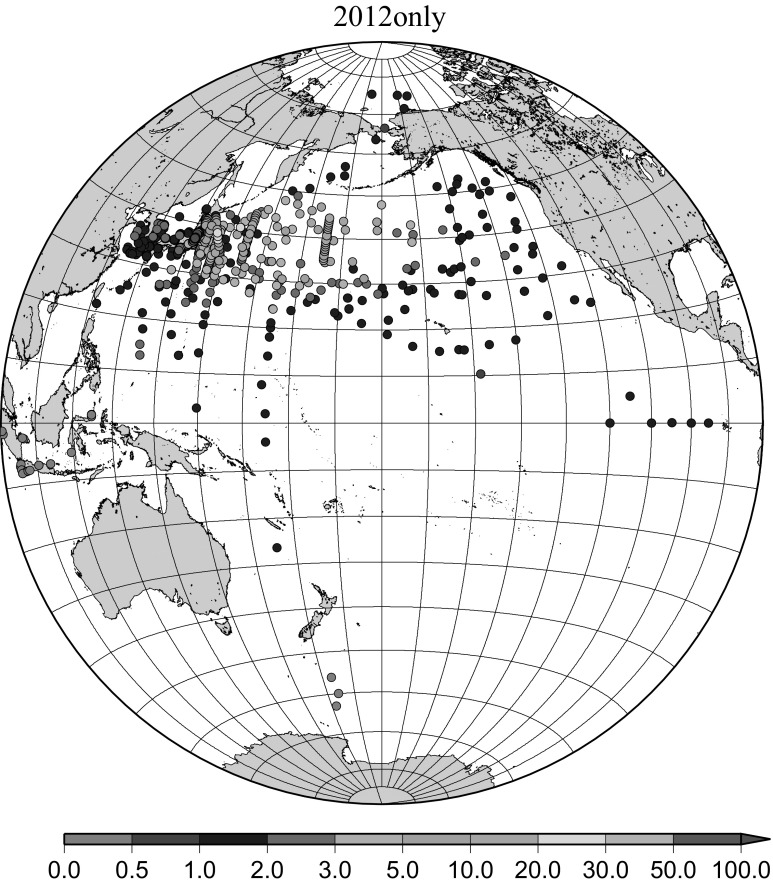
Horzontal distribution of ^137^Cs activity concentration in the North Pacific in 2012 only. Unit: Bq m^−3^

**Fig. 14:**
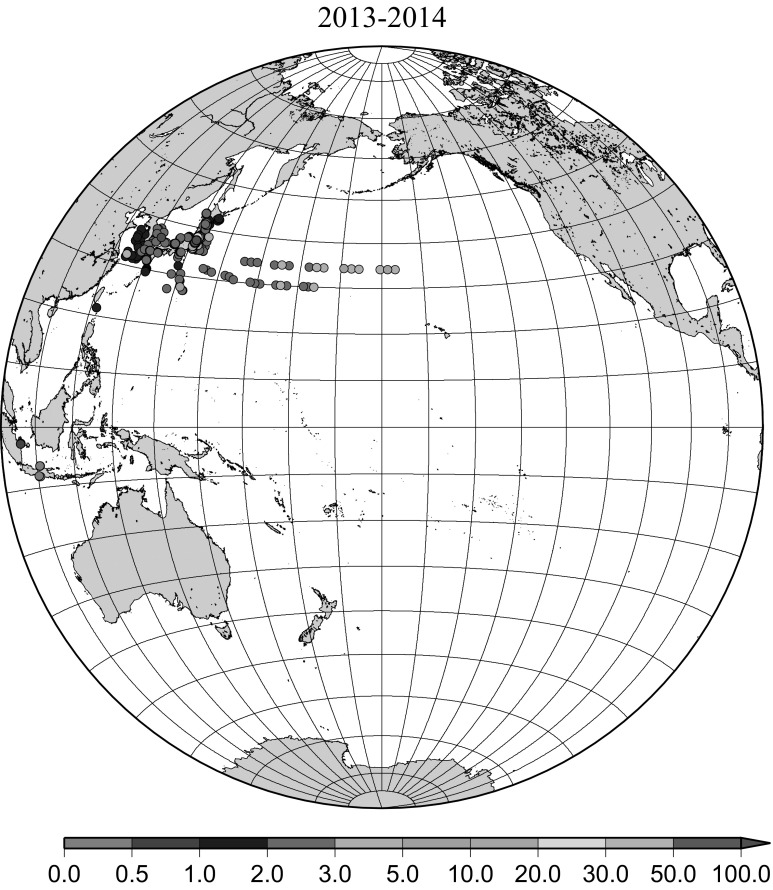
Horzontal distribution of ^137^Cs activity concentration in the North Pacific in 2013–2014. Unit: Bq m^−3^

**Fig. 15:**
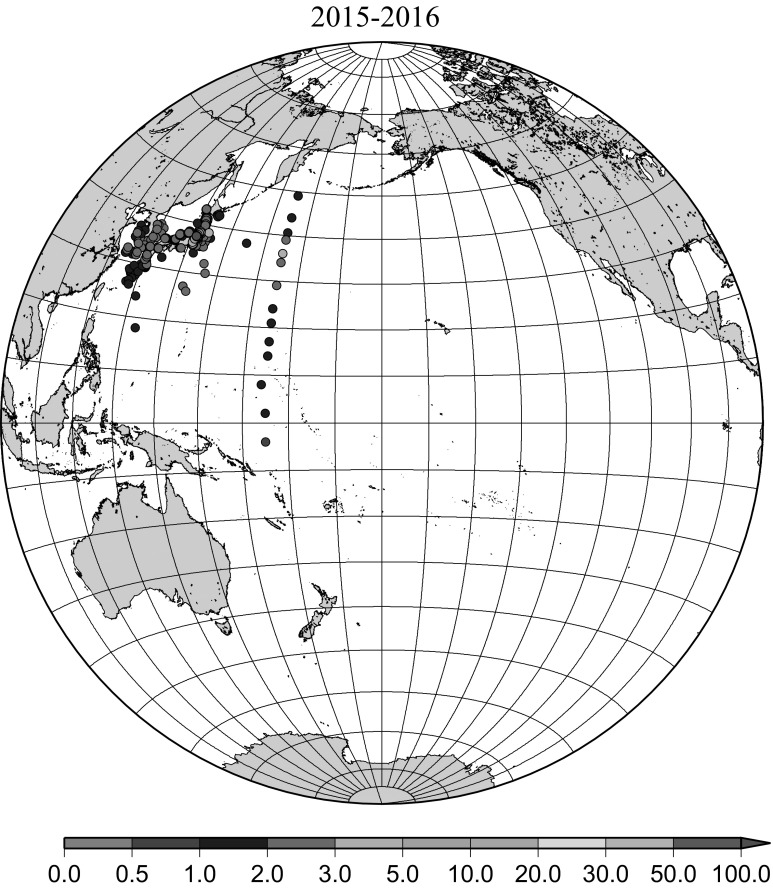
Horzontal distribution of ^137^Cs activity concentration in the North Pacific in 2015–2016. Unit: Bq m^−3^

Detailed horizontal distribution of ^137^Cs concentrations in surface seawater in the Pacific Ocean from 1957 to 2011 are shown in Figs. 5, 6, 7, 8, 9, 10, 11, 12, 13, 14 and 15 for the periods, 1956–1962, 1963–1969, 1970–1979, 1980–1989, 1986 only, 1990–1999, 2000–2010, 2011 only, 2012 only, 2013–2014, 2015–2016, respectively. And in this case these data were not radioactive decay corrected. Because the data density was not high enough for drawing contour lines at wide area in the Pacific Ocean, the ^137^Cs data were plotted with colour scale of corresponding without decay correction.

The ^137^Cs in surface seawater in the North Pacific Ocean have originated mainly from the weapons tests at PVG in the North Pacific Ocean in early 1950s and global fallout in the 1950s and 1960s as described above. Distribution of radioactive contamination in early 1950s was characterized by local fallout very close to the test sites as initial pattern, then transported by strong surface current close to the test site namely the north equatorial current towards west (Fig. 2). Then the radioactivity was transported by Kuroshio Current and Kuroshio extensions which consist a part of subtropical gyre which goes clockwise, goes west first then turn to north then eastward (Fig. 3).

The effect of global fallout was large in 1956–1963 as shown in Fig. 5. The highest concentrations were found in the western North Pacific Ocean (30–45°N, 135–155°E) These areas correspond to crossovers of areas where larger precipitation amounts and areas where higher stratosphere–troposphere exchange was expected as already stated [4, 25]. The effect of local fallout originating from nuclear weapon tests carried out at PVG around Bikini Atoll, can clearly be seen in the western part of the equatorial Pacific Ocean as shown in Figs. 2, 3 and 5 [26]. Global distribution of ^137^Cs activity concentration in surface water followed global fallout pattern until early 1960s which showed mid latitude maxima in both Northern and Southern Hemispheres due to geographical distribution of areas where stratosphere and troposphere exchange is dominant. Thereafter, meridional gradient of ^137^Cs activity in surface water tend to be smaller because the global circulation of surface water homogenised ^137^Cs activity. The higher concentrations moved to the mid of eastern North Pacific Ocean in the 1960s (Fig. 6). As shown in Fig. 7, the difference of ^137^Cs concentrations in surface seawater between mid-latitudes and the equatorial region in the 1970s became smaller [27]. It is obvious that both research activity and monitoring activity in the eastern and central Pacific Ocean became low in 1980s, 1990s and 2000s while Japanese activity had been keeping in these periods. In the Pacific Ocean, latitudinal difference in ^137^Cs activity concentrations in surface seawater became small in 1970s, 1980s, 1990s and 2000s as shown in Figs. 6, 7, 8, 10 and 11. The impact of the Chernobyl accident on the marine radioactivity in the Pacific Ocean was negligible (but measurable) in the western North Pacific Ocean especially in mid latitude (Fig. 9). During the period from 2000 to 2010 (Fig. 11), latitudinal difference in ^137^Cs concentrations in surface seawater became small, and concentrations of ^137^Cs tended to be almost homogeneous in the surface seawater of the global ocean, we however can see weak mid-latitude maximum of ^137^Cs concentration still in 2000s.

The impact of the Fukushima accident on the marine radioactivity in the Pacific Ocean was significant because the accident site located at coastal area of Japanese island and faced to the North Pacific Ocean [28, 29]. Radioactive substances were released from the TEPCO Fukushima Dai-ichi nuclear power plant (FNPP1) accident into the environment beginning on March 11, 2011. Because a large portion of released radioactivity from the FNPP1, which included both radiocesium and radioiodine, might be soluble, the movement of seawater can be responsible for large-scale transport. Because the FNPP1 is located in a coastal region with strong coastal currents along the coast in a north–south direction, radioactivity discharged or deposited in the coastal region can be advected by these strong coastal currents and is less affected by diffusion. In this region, the Kuroshio Current comes from the south, originating far from Japan, and the Oyashio current comes from north, originating near the Aleutian Islands and Kuril Islands [28]. Transport of FNPP1 derived radiocaesium to the south was initially dominant before eastward propagation resulting from the Kuroshio and Kuroshio extension became dominant. In April–June 2011, radiocaesium released from the FNPP1 to the atmosphere was transported to the northeast from Japan. Therefore, the radiocaesium activity concentration in the surface water was high at high latitudes between 35 deg. N and 45 deg. N in the western North Pacific Ocean while the radiocaesium activity concentration in the surface water was not high south of 30 deg. N latitude as shown in Fig. 12. In addition, relatively high radiocaesium activities were observed due to locally deposited radiocaesium at several places in the North Pacific Ocean (Fig. 12) [15]. FNPP1 radiocaesium spread eastward in the surface water across the mid-latitude North Pacific with a speed of 8 cm s^−1^ until March 2012 and 3.5 cm s^−1^ from March 2012 through August 2014 [15]. In Fig. 13 for horizontal distribution of ^137^Cs activity concentration in 2012, main body of Fukushima derived radiocaesium was around 40 deg. N latitude in the central Pacific Ocean with local maximum very close to the Fukushima accident site which indicate continuous leaking from the site [30]. Thereafter the Fukushima derived radiocaesium reached the western coast of the US continent in 2014/15 [31, 32]. In 2013/2014 and 2015/2016, observed/published ^137^Cs activity concentration data is limited in the western North Pacific Ocean. In general we can still see weak mid latitude maximum and local maximum very close to the Fukushima accident site.

### Time-latitude diagram analyses, Hovmoller diagram, of ^137^Cs activity concentration in surface water

**Fig. 16:**
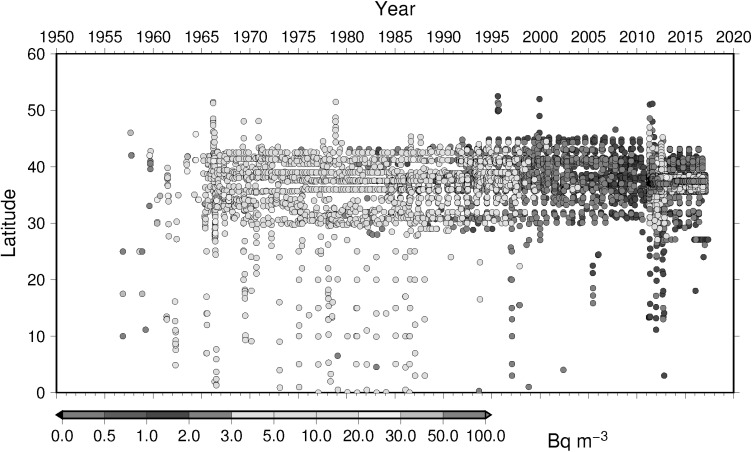
Hovmoller diagram of ^137^Cs in surface water for a longitude band between 130 deg. E and 160 deg. E in the North Pacific until 2016

**Fig. 17:**
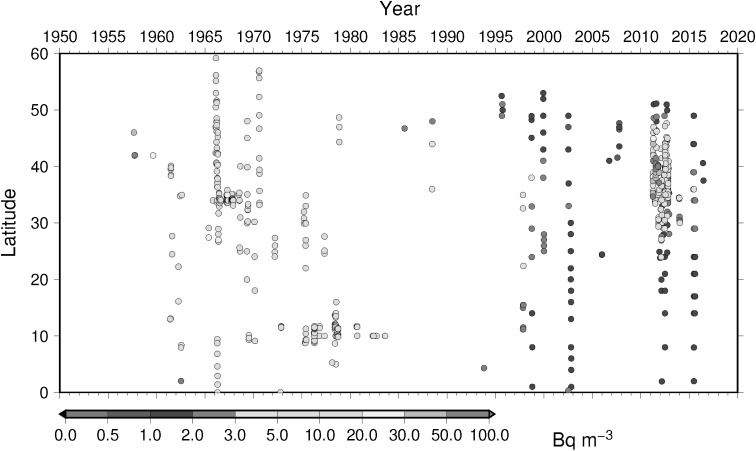
Hovmoller diagram of ^137^Cs activity concentration in surface water for a longitude band between 150 deg. E and 170 deg. E in the North Pacific until 2016

**Fig. 18:**
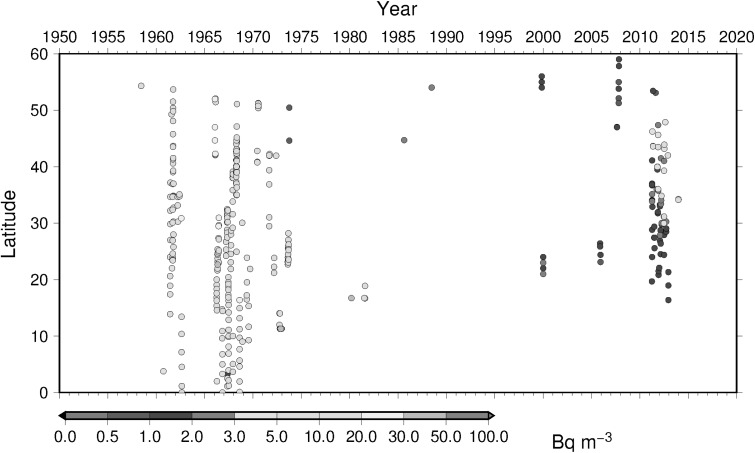
Hovmoller diagram of ^137^Cs activity concentration in surface water for a longitude band between 180 deg. E and 160 deg. W in the North Pacific until 2016

**Fig. 19:**
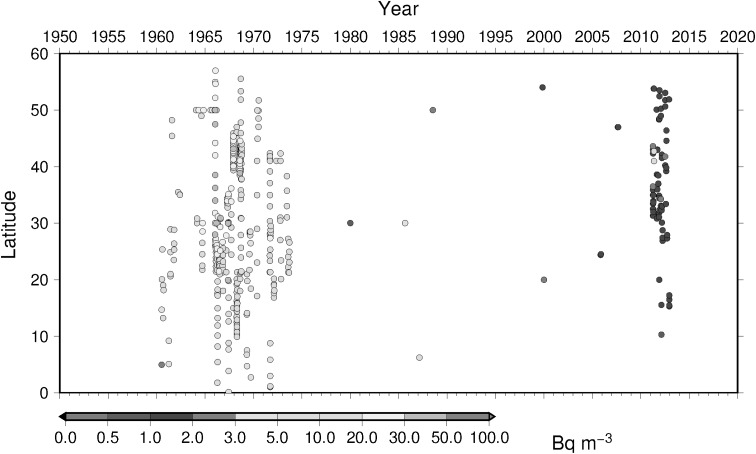
Hovmoller diagram of ^137^Cs activity concentration in surface water for a longitude band between 160 deg. W and 140 deg. W in the North Pacific until 2016

**Fig. 20:**
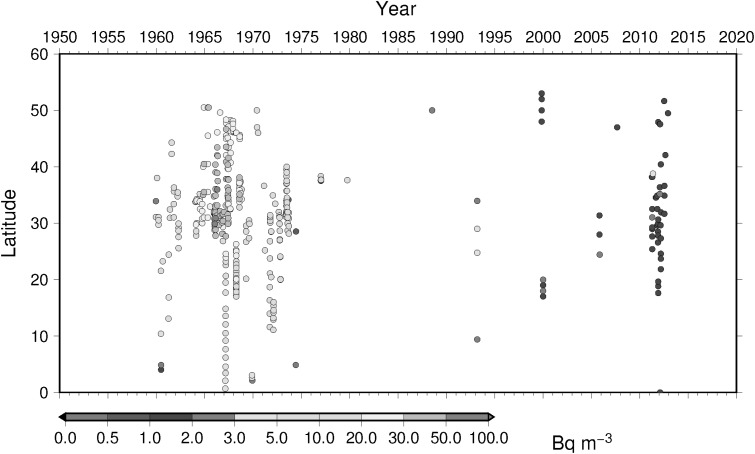
Hovmoller diagram of ^137^Cs activity concentration in surface water for a longitude band between 140 deg. E and 120 deg. E in the North Pacific until 2016

To understand temporal change of ^137^Cs activity concentration in surface water in the North Pacific Ocean, Hovmoller diagram of ^137^Cs activity concentration in surface water for 5 longitude band from west to east as zone 1 through zone 5 (Table 4) are shown in Figs. 16, 17, 18, 19 and 20. In zone 1 (Fig. 16) most western region in the western North Pacific Ocean between 130 deg. E and 160 deg. E, it is clear that impact of local fallout at PVG tests in 1950s was clear at lower latitude around 10 deg. N. It is also obvoius that north-east wards movement of local fallout around 25 deg. N–40 deg. N. In early 1960s, ^137^Cs activity concentration derived from global fallout was observed at levels of 20–50 Bq m^−3^ between 30 deg. N to 40 deg. N. In late 1960s and 1980s the ^137^Cs activity concentration was around 5–20 Bq m^−3^. We can see weak Chernobyl derived ^137^Cs in 1986 at around 40 deg. N. Until the Fukushima accident in 2011, ^137^Cs activity concentration in the mid latitude in zone 1 most western region in the western North Pacific Ocean tended to decrease and down to 1–2 Bq m^−3^ before the Fukushima accident. The impact of Fukushima accident is obvious that since Fukuhsima site locates at around 37 deg. N ^137^Cs activity concentration in surface water showed a maximum at 37 deg. N and spreaded to north and south bu both atmospheric dispersion and direct discharge from the site. The contaminated water moved eastward and north–south extension of ^137^Cs activity concentration in surface water shrinked as shown in Fig. 16. In zone 2 between zone 1 and international dataline, due to relatively long distance from the source region of local fallout and global fallout and less number of observed data as shown in Fig. 17, weak mid latitude maximum before Fukushima accident was a feature. After the Fukushima accident, a maximum of ^137^Cs activity concentration was observed relatively high latitude at around 40 deg. N because of north-east atmospheric transport of radiocaesium. In zone 3, mid of theNorth Pacific Ocean, a similar pattern was observed as well as observed in zone 2 except after Fuksuhima accident. In this mid North Pacific region, maximum of ^137^Cs activity concentration in surface water was observed higher latitude between 40 deg. N and 50 deg. N.

**Table 4:** Longitudinal zones for Hovmoller diagrams of ^137^Cs activity concentration in surface water in the Pacific Ocean

Zone name	Longitude west	Longitude east
Zone 1	130 E	160 E
Zone 2	150 E	170 E
Zone 3	180 E	160 W
Zone 4	160 W	140 W
Zone 5	140 W	120 W

In the zone 4 and 5, eastern North Pacific Ocean, the behavior of global fallout ^137^Cs activity concentration in surface water was quite different from those at the western Paficic Ocean. The ^137^Cs activity concentration in surface water reached 70 Bq m^−3^ which was higher rather than those observed in wester North Pacific Ocean. Based on modeling study [33] and previous study [34], the contaminated surface water produced in the western Pacific Ocean and they advected to east then global fallout had been continued at the region where mixed layer depth was shallower, finally ^137^Cs activity concentration in surface water increased rather than those in originally advected from west.

### Surface to ocean interior, in case of Fukushima accident and global fallout

**Fig. 21:**
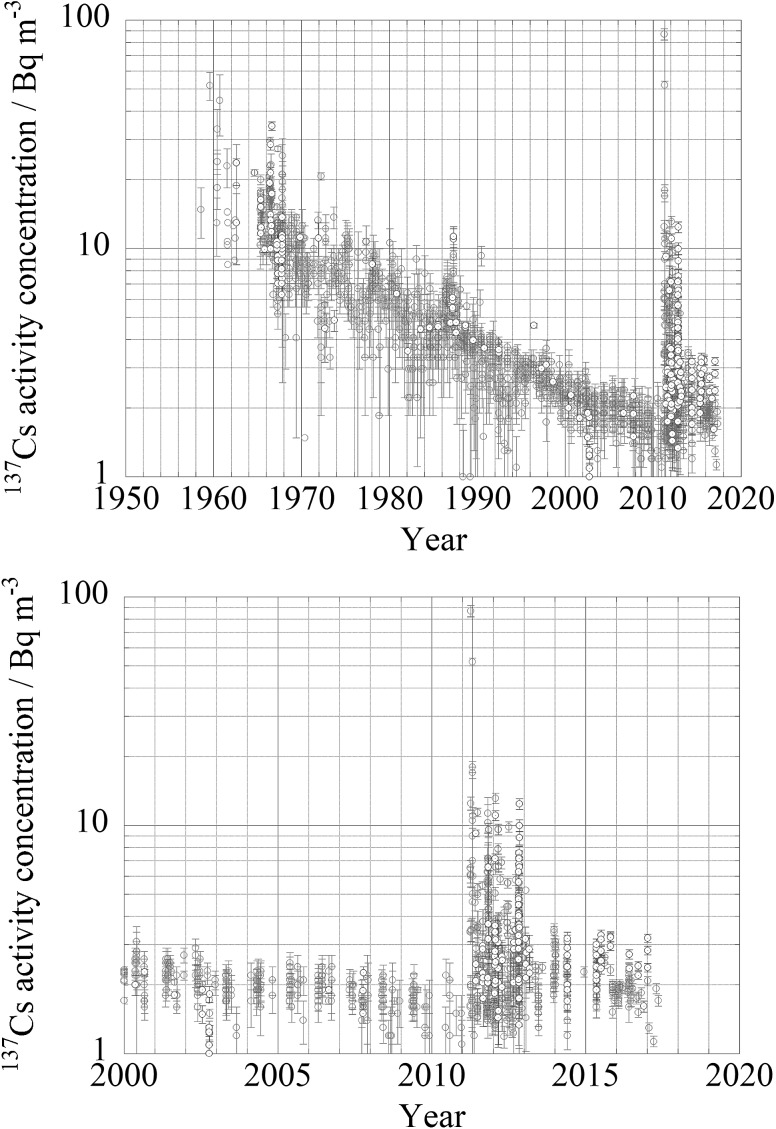
Temporal variation of ^137^Cs activity concentration at mid latitude, 25 deg. N to 35 deg. N, in the western North Pacific Ocean until 2016. Red circle: ^137^Cs activity concentration in surface layer shallower than 10 m. Blue circle: ^137^Cs activity concentration in a layer between 100 and 220 m depth. (Color figure online)

**Fig. 22:**
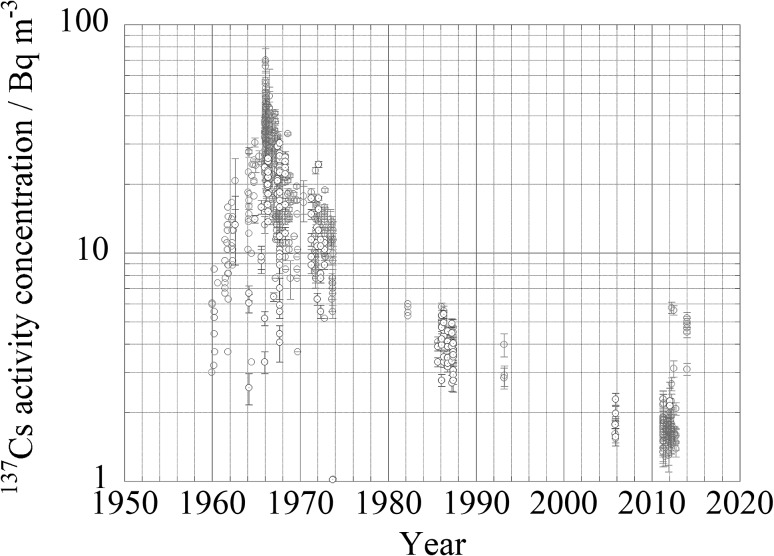
Temporal variation of ^137^Cs activity concentration at mid latitude, 25 deg. N to 35 deg. N, in the eastern North Pacific Ocean until 2016. Red circle: ^137^Cs activity concentration in surface layer shallower than 10 m. Blue circle: ^137^Cs activity concentration in a layer between 100 and 220 m depth. (Color figure online)

In case of Fukushima accident we have much data to discuss about transport processes from ocean surface to ocean interior. Beneath the ocean’s surface, in June 2012, the ^134^Cs activity reached a maximum of 6.12 ± 0.50 Bq m^−3^ at a depth of 151 m (potential density, *σ*_*θ*_ = 25.3 kg m^−3^) at 29°N, 165°E. This subsurface maximum, which was also observed along 149°E, might reflect the southward transport of the FNPP1 radiocaesium in association with the formation and subduction of Subtropical Mode Water (STMW). In June 2012, between 34°N and 39°N along 165°E, the ^134^Cs activity showed a maximum of approximately *σ*_*θ*_ = 26.3 kg m^−3^, corresponding to the Central Mode Water (CMW). The ^134^Cs activity was higher in the CMW than in any of the surrounding waters, including the STMW. These observations also indicated that the most efficient pathway by which FNPP1 radiocaesium was introduced into the ocean on a 1-year time scale was through CMW formation and subduction [15]. Kaeriyama et al. [35] and Kumamoto et al. [36, 37] already reported about impact of subduction of Fukushima derived radiocaesium into Sub tropical Mode water. As a results of subduction, subsurface/mid depth maximum occurred and this situation might be kept long time for several decades [38]. As shown in Fig. 21a, ^137^Cs activity concentration in sub surface water at 100–200 m depth in the western North Pacific Ocean was similar or higher rather than those in surface layer jst after larger injection on ocean surface in 1960s, in 1986 and in 2012–2016 after the Fukushima accident. As shown in Fig. 21b, effect of subduction was obvious in 2012. ^137^Cs activity concentration in sub surface water at 100–200 m depth reached 10 Bq m^−3^ while that in surface water was around several Bq m^−3^. In 2015 and 2016, ^137^Cs activity concentration in sub surface water at 100–200 m depth exceeded 2 Bq m^−3^ while that in surface water was around 1–1.5 Bq m^−3^. In contrast to western North Pacific Ocean, as shown in Fig. 22 for the eastern North Pacific Ocean, ^137^Cs activity concentration in sub surface water at 100–200 m depth was similar or lower than those in surface water because subduction processes are quite different at both side. The details of behavior of ^137^Cs activity concentration will be discussed in part II of this article (Aoyama et al. in preparation)

## Conclusions

Temporal variation of source term of radiocaesium in the Pacific Ocean was presented as full deposition history at Tokyo/Tukuba, Japan since 1945 until 2016. HAM database and its update which include were used in this study to present whole history of radiocaesium transport in surface layer in the interested region. The HAM database and its update, which constructed from 157 literatures and Japanese governmental monitoring data accumulating since 1953–2016 and included 55,811 records for ^137^Cs activity concentration data. Based on the observed data in updated HAM database, long range transport of radiocaesium derived from local fallout occurred early 1950s, global fallout which occurred mainly late 1950s and early 1960s and the Fukushima accident occurred in 2011 were investigated and presented for ocean surface in the Pacific Ocean. Since both the main local/global fallout regions and injection of radiocaesium by Fukushima accident occurred in the western North Pacific and constrain of surface current systems which governed surface transport processes were subtropical gyre and subarctic gyre, radiocaesium transport in surface water in the mid latitude was characterized as rapid eastward transport along Kuroshio and Kuroshio Extension. Behaviors were similar and repeated for local/global fallout and Fukushima derived radiocaesium. A part of radiocaesium transported/deposited/injected in the mid latitude subducted into ocean interior and the radiocaesium activity concentrations were kept higher rather than those in surface water.

## Electronic supplementary material

Below is the link to the electronic supplementary material.

Supplementary material 1 (DOCX 28 kb)

Supplementary material 2 (XLSX 13 kb)

Supplementary material 3 (XLS 32 kb)

Supplementary material 4 (XLS 74 kb)

Supplementary material 5 (XLS 77 kb)
