# Supplementary material for: Long-range transport of radiocaesium derived from global fallout and the Fukushima accident in the Pacific Ocean since 1953 through 2017—Part I: Source term and surface transport
Source: J Radioanal Nucl Chem. 2018 Oct 19;318(3):1519–42. doi: 10.1007/s10967-018-6244-z (PMC6267156; doi:10.1007/s10967-018-6244-z)
Supplement: Supplementary file 1 — Supplementary material 1 (DOCX 28 kb) [file 10967_2018_6244_MOESM1_ESM.docx]

Table S1 Data sources and references for figures 1, 2, and 5 to 22.

Aoyama, M., et al. (2013), 'Surface pathway of radioactive plume of TEPCO Fukushima NPP1 released 134Cs and 137Cs', *Biogeosciences,* 10 (5), 3067-78.

Aoyama, M., et al. (2001), '137Cs activity in surface water in the western North Pacific', *Journal of Radioanalytical and Nuclear Chemistry,* 248 (3), 789-93.

Aoyama, M., et al. (2011), 'Cross equator transport of 137Cs from North Pacific Ocean to South Pacific Ocean (BEAGLE2003 cruises)', *Progress in Oceanography,* 89 (1-4), 7-16.

Aoyama, Michio and Hirose, Katsumi (1995), 'The temporal and spatial variation of 137Cs concentration in the Western North Pacific and its marginal seas during the period from 1979 to 1988', *Journal of Environmental Radioactivity,* 29 (1), 57-74.

Aoyama, Michio, et al. (2008), 'Water masses labeled with global fallout137Cs formed by subduction in the North Pacific', *Geophysical Research Letters,* 35 (1), L01604.

Aoyama, Michio, et al. (2016), '134Cs and 137Cs in the North Pacific Ocean derived from the March 2011 TEPCO Fukushima Dai-ichi Nuclear Power Plant accident, Japan. Part one: surface pathway and vertical distributions', *Journal of Oceanography,* 72 (1), 53-65.

Aoyama, Michio, et al. (2018), 'Radiocaesium derived from the TEPCO Fukushima accident in the North Pacific Ocean: Surface transport processes until 2017', *Journal of Environmental Radioactivity,* 189, 93-102.

Bourlat, Y., et al. (1996), '90Sr, 137Cs and 239+240Pu in world ocean water samples collected from 1992 to 1994', in P. Guéguéniat, P. Germain, and H. Métivier (eds.), *Radionuclides in the Oceans Inputs and Inventories* (Cherbourg, France: Institut de Protection et de Surete Nucleaire), 75-93.

Bowen, V.T. (1982), 'Transuranic behaviour in marine environment', (IAEA-TECDOC-265: Woods Hole Oceanographic Institution), 129-55.

Broecker, W. S. and Simpson, H. J. (1968), 'A summary of Lamont Sr-90 and Cs-137 measurements on ocean water samples. HASL-197', (US Atomic Energy Commission), I204-I26.

Broecker, Wallace S., Bonebakker, Erno R., and Rocco, Gregory G. (1966), 'The vertical distribution of cesium 137 and strontium 90 in the oceans, 2', *Journal of Geophysical Research,* 71 (8), 1999-2003.

Buesseler, K. O., et al. (2012), 'Fukushima-derived radionuclides in the ocean and biota off Japan', *Proc Natl Acad Sci U S A,* 109 (16), 5984-8.

Delfanti, R., et al. (2000), 'IAEA CRP "Worldwide Marine Radioactivity"', (Italy).

Donaldson, L. R., (1956) Survey of radioactivity in the sea near Bikini and Eniwetok Atolls, UWFL-46, Washington. Warship Walton data is in this report.

Domanov, MM, et al. (2004), 'Anthropogenic radionuclides and the radioecological situation in the sea of Japan', *Okeanologiya,* 44 (3), 380-88.

Folsom, T. R. (1979), 'Summary of Cs-137 Concentrations Measured at Scripps Institution in N. Pacific Surface Waters', *Environmental Measurements Laboratory Report* (EML-356; New York), 157-250.

Folsom, T. R. and Mohanrao, G. J. (1960), 'Measurement of Fallout Cesium in the Pacific Ocean and in Terrestrial Effluents likely to alter Coastal Waters', *Journal of Radiation Research,* 1 (2), 150-54.

Folsom, T. R., Mohanrao, G. J., and Winchell, P. (1960), 'Fallout caesium in surface sea water off the California coast (1959-60) by gamma-ray measurements', *Nature,* 187, 480-82.

Folsom, T. R., et al. (1968), 'Distributions of Cs-137 in the Pacific. HASL-197', (1968/07/01 edn.: Atomic Energy Commission), I95-I105.

Folsom, T. R., et al. (1975), 'Recent improvements in methods for concentrating and analyzing radiocesium in sea water', *Journal of radiation research,* 16 (1), 19-27.

Folsom, T.R., et al. (1970), 'Some concentration of Cs137 at moderate depths in the Pacific 1965-1968, HASL-217 ', (Scripps Institution of Oceanography, University of California), I9-I129.

Hirose, K., Sugimura, Y., and Aoyama, M. (1992), 'Plutonium and 137Cs in the western North Pacific: Estimation of residence time of plutonium in surface waters', *International Journal of Radiation Applications and Instrumentation. Part A. Applied Radiation and Isotopes,* 43 (1-2), 349-59.

Hirose, K., et al. (2001), *Long-term trends of plutonium fallout observed in Japan. In: Kudo A (ed) Radioactivity in the environment Vol 1. pp 251–266. Plutonium in the environment proceedings of the second international symposium, Osaka, Japan, November 9–12, 1999* (Amsterdam: Elsevier).

Hirose, K., et al. (2002), 'Plutonium isotopes in the Sea of Japan', *Journal of Radioanalytical and Nuclear Chemistry,* 252 (2), 293-99.

Hirose, K., et al. (1999), 'Anthropogenic radionuclides in seawater in the East Sea/Japan Sea: results of the first-stage Japanese-Korean-Russian expedition', *J Environ Radioact,* 43.

Hong, G. H., et al. (1999), 'Artificial radionuclides in the East Sea (Sea of Japan) proper and Peter the Great Bay', *Marine Pollution Bulletin,* 38 (10), 933-43.

Hydrographie , Bundesamt für Seeschifffahrt und 'BSH1997', (Bundesamt für Seeschifffahrt und Hydrographie ).

Ikeuchi, Y., et al. (1999), 'Anthropogenic radionuclides in seawater of the Far Eastern Seas', *Science of The Total Environment,* 237–238 (0), 203-12.

Inoue, M., et al. (2012), 'Low levels of 134Cs and 137Cs in surface seawaters around the Japanese Archipelago after the Fukushima Dai-ichi Nuclear Power Plant accident in 2011', *Geochem. J,* 46, 311-20.

Ito, T., et al. (2003), 'Anthropogenic radionuclides in the Japan Sea: their distributions and transport processes', *Journal of Environmental Radioactivity,* 68 (3), 249-67.

Japan Atomic Energy Commission (1959) Houshanou Chousa no Tenbou, in Japanese. NORPAC data is in this report.

Kaeriyama, H., et al. (2013), 'Direct observation of 134Cs and 137Cs in surface seawater in the western and central North Pacific after the Fukushima Dai-ichi nuclear power plant accident', *Biogeosciences Discussions,* 10 (2), 1993-2012.

Kaeriyama, Hideki, et al. (2014), '134 Cs and 137 Cs in seawater around Japan after the Fukushima Daiichi Nuclear Power Plant accident'.

Kameník, J, et al. (2013), 'Cesium-134 and 137 activities in the central North Pacific Ocean after the Fukushima Dai-ichi Nuclear Power Plant accident', *Biogeosciences,* 10 (9), 6045-52.

Kang, Dong-Jin, et al. (1997), 'Distribution of 137Cs and 239, 240Pu in the surface waters of the East Sea (Sea of Japan)', *Marine Pollution Bulletin,* 35 (7-12), 305-12.

Kim, C. K., et al. (2012), 'Radiological impact in Korea following the Fukushima nuclear accident', *Journal of environmental radioactivity,* 111, 70-82.

KINS/ER-092, Vol.7 to Vol.12: Marine Environmental Radioactivity Survey 2011, KOREA INSTITUTE OF NUCLEAR SAFETY ..And this report series until for 2016.

Kumamoto, Yuichiro, et al. (2015), 'Impact of Fukushima-derived radiocesium in the western North Pacific Ocean about ten months after the Fukushima Dai-ichi nuclear power plant accident', *Journal of environmental radioactivity,* 140, 114-22.

Kumamoto, Yuichiro, et al. (2017), 'Fukushima-derived radiocesium in the western North Pacific in 2014', *Journal of Radioanalytical and Nuclear Chemistry,* 311 (2), 1209-17.

Livingston, H.D. (2000).

Livingston, Hugh D., et al. (1984), 'Vertical profile of artificial radionuclide concentrations in the central Arctic Ocean', *Geochimica et Cosmochimica Acta,* 48 (11), 2195-203.

Livingston, Hugh D., et al. (1985), 'Fallout nuclides in Atlantic and Pacific water columns: GEOSECS data', *WHOI Technical Reports*, 75.

Livingston, H.D., Bowen, V.T., Casso, S.A., Volchok, H.L., Noshkin., V.E., Wong, K.M., and Beasley, T.M. (1985) Fallout Nuclides in Atlantic and Pacific Water Columns: GEOSECS Data. Woods Hole Oceanographic Institute Tech. Rep., WHOI-85-19.

MSA1965-MSA2000: Hydrographic Department, Maritime Safety Agency, Ministry of Transport (1967) Annual Reports of Radioactivity Survey for 1965 [in Japanese]. And this report series until for 2010.

MERI1985-MERI2016: Report of Japanese Governmental radioactivity monitoring in marine environment for 1985 to 2016.

Miyake et al., (1955) Bikini kaiiki ni okeru jinnkou houshnaou no Bunpu to sono kaiyougakuteki kousatu, Kagaku, 24, 601-605, in Japanese. R/V shunnkotu-maru data in this article.

Miyake, Y. (1963), 'Artificial radioactivity in the Pacific Ocean. In Radioactive Tracers in Oceanography', *IUGG Monograph* (20: International Union of Geodesy and Geophysics), 21-30.

Miyake, Y., Saruhashi, K., and Katsuragi, Y. (1960), 'Strontium 90 in western North Pacific surface waters', *Papers in Meteorology and Geophysics,* 11, 188–91.

Miyake, Y., et al. (1962), 'Penetration of 90Sr and 137Cs in deep layers of the Pacific and vertical diffusion rate of deep water', *Journal of radiation research,* 3-4, 141-47.

Miyake, Y., et al. (1988), 'Contents of 137Cs, plutonium and americium isotopes in the Southern Ocean waters', *Papers in Meteorology and Geophysics,* 39 (95-113).

Miyake, Yasuo, et al. (1961), 'Cesium 137 and strontium 90 in sea water', *Journal of Radiation Research,* 2 (1), 25-28.

Miyao, Takashi, et al. (1998), 'Temporal variation of 137Cs and 239,240Pu in the Sea of Japan', *Journal of Environmental Radioactivity,* 40 (3), 239-50.

Nagaya, Y. and Nakamura, K. (1970), 'A study on the vertical transport of 90Sr and 137Cs in the surface waters of the seas around Japan', *Journal of Radiation Research,* 11 (1), 32-43.

Nagaya, Y., Shiozaki, M., and Seto, Y. (1965), 'Some fallout radionuclides in deep waters around Japan', *Journal of radiation research,* 6 (1), 23-31.

Nagaya, Yutaka and Nakamura, Kiyoshi (1976), '90Sr and 137Cs contents in the surface waters of the adjacent seas of Japan and the North Pacific during 1969 to 1973', *Journal of the Oceanographical Society of Japan,* 32 (5), 228-34.

--- (1981), 'Artificial radionuclides in the western Northwest Pacific (I)90Sr and137Cs in the deep waters', *Journal of the Oceanographical Society of Japan,* 37 (3), 135-44.

--- (1984), '239, 240Pu, 137Cs and 90Sr in the central North Pacific', *Journal of the Oceanographical Society of Japan,* 40 (6), 416-24.

--- (1987), 'Artificial radionuclides in the western Northwest Pacific (II):137Cs and239,240Pu inventories in water and sediment columns observed from 1980 to 1986', *Journal of the Oceanographical Society of Japan,* 43 (6), 345-55.

--- (1993), 'Distributions and mass-balance of 239,240Pu and 137Cs in the northern North Pacific', in T. Teramoto (ed.), *Deep Ocean Circulation: Physical and Chemical Aspects* (Elsevier Oceanography Series; Amsterdam: Elsevier), 157-67.

Noshikin, V.E.(1974a), Plutonium oxidation state distributions in the Pacific Ocean during 1980-1981, xxx

Noshkin, V.E., Wong, K.M., Eagle, R.J., Gatrousis, C.(1974b) Transuranics at Pacific Atolls. I. Concentrations in the Waters at En(T. R. Folsom et al. 1975)ewetak and Bikini, WOMARS

Noshkin, V.E. (1999) Concentrations of Radionuclides in Some Seawater and Sediment Samples from the Equatorial Pacific with Emphasis on Samples from the Western Pacific Ocean. Preliminary report prepared for the 1st Coordinated Research Project on Worldwide Marine Radioactivity Studies.

Noshkin, V.E., Wong, K.M., Jokela, T.A., Eagle, R.J., and Brunk, J.L. (1978) Radionuclides in the Marine Environment Near the Farallon Island. UCRL-52381. pp. 16.

Noshkin, V.E. (1987) Personal communication.

Noshkin, V.E. (1999) Concentrations of Radionuclides in Some Seawater and Sediment Samples from the Equatorial Pacific with Emphasis on Samples from the Western Pacific Ocean. Preliminary report prepared for the 1st Coordinated Research Project on Worldwide Marine Radioactivity Studies.

Noshkin, V.E. (2000) Personal communication.

Noshkin, V.E., Eagle, R.J., and Wong, K.M. (1976) Plutonium levels in Kwajalein Lagoon. Nature 262, 745–748.

Pillay, K.C., Smith, R.C., and Folsom, T.R. (1964) Plutonium in the marine environment. Nature 203, 568–571.

Povinec, P. P., et al. (2011), '137Cs water profiles in the South Indian Ocean – An evidence for accumulation of pollutants in the subtropical gyre', *Progress in Oceanography,* 89 (1–4), 17-30.

Povinec, P. P., et al. (2013), 'Cesium, iodine and tritium in NW Pacific waters &ndash; a comparison of the Fukushima impact with global fallout', *Biogeosciences,* 10 (8), 5481-96.

Saruhashi, K., et al. (1975), '90Sr and 137Cs in the Pacific waters', *Records of Oceanographic Works in Japan,* 13, 1-15.

Science and Technology Agency, Japan,(1995) Investigation of environmental radioactivity in waste dumping areas of the Far Eastern seas: results from the first Japanese-Kore-Russian joint expedition 1994, white paper.

Shirasawa, T. H. and Schuert, E. A. (1968), 'Fallout radioactivity in the North Pacific Ocean: data compilation of Sr-90 and Cs-137 concentrations in seawater. HASL-197', *HASL Rep* (1968/07/01 edn.: Oak Ridge Tn : Us Atomic Energy Commission), I1-I66.

Suseno, H. and Prihatiningsih, W. R. (2014), 'Monitoring 137Cs and 134Cs at marine coasts in Indonesia between 2011 and 2013', *Mar Pollut Bull,* 88 (1-2), 319-24.

Suseno, Heny, Wahono, Ikhsan Budi, and Muslim (2015), 'Radiocesium monitoring in Indonesian waters of the Indian Ocean after the Fukushima nuclear accident', *Marine Pollution Bulletin,* 97 (1), 539-43.

U.S. Atomic Energy Commission. Health and Safety Laoratory (1956) NYO4656. Operation Troll data is in the report.

U.S. Atomic Energy Commission. Health and Safety Laoratory (1958) Pacific Sea Water samples, reprint from the collection of the University of California Libraries, 2838695R00111.

Wong, K.M. (1971).

Wong, Kai M., et al. (1992), 'Radionuclide concentrations, fluxes, and residence times at Santa Monica and San Pedro Basins', *Progress in Oceanography,* 30 (1-4), 353-91.

Yamada, M. (1997), '239+240Pu and137Cs concentrations in salmon (Oncorhynchus keta) collected on the Pacific coast of Japan', *Journal of Radioanalytical and Nuclear Chemistry,* 223 (1), 145-48.

Yamada, M., Aono, T., and Hirano, S. (1996), '239+240Pu and137Cs distributions in seawater from the Yamato Basin and the Tsushima Basin in the Japan Sea', *Journal of Radioanalytical and Nuclear Chemistry,* 210 (1), 129-36.

Yamada, Masatoshi and Wang, Zhong-Liang (2007), '137Cs in the western South Pacific Ocean', *Science of The Total Environment,* 382 (2–3), 342-50.

Yamada, Masatoshi, Zheng, Jian, and Wang, Zhong-Liang (2006), '137Cs, 239 + 240Pu and 240Pu / 239Pu atom ratios in the surface waters of the western North Pacific Ocean, eastern Indian Ocean and their adjacent seas', *Science of The Total Environment,* 366 (1), 242-52.

Zhou, Peng, et al. (2018), 'Radioactive status of seawater and its assessment in the northeast South China Sea and the Luzon Strait and its adjacent areas from 2011 to 2014', *Marine Pollution Bulletin,* 131, 163-73.
